# Supplementary material for: Consortium of Lactobacillus crispatus 2029 and Ligilactobacillus salivarius 7247 Strains Shows In Vitro Bactericidal Effect on Campylobacter jejuni and, in Combination with Prebiotic, Protects Against Intestinal Barrier Dysfunction
Source: Antibiotics (Basel). 2024 Nov 28;13(12):1143. doi: 10.3390/antibiotics13121143 (PMC11672454; doi:10.3390/antibiotics13121143)
Supplement: Supplementary file 1 [file antibiotics-13-01143-s001.zip › antibiotics-3298245-supplementary.pdf]

Supplementary material

Table S1. Human primers used for qRT-PCR analysis of host immune response and homeostasis regulation by BLLT1

Primer	Sense primer	Antisense primer	Reference
TLR4	TGCACAGGACAGAACATCTCTGGA	AGCTCCTGCAGGGTATTCAAGTGT	[211]
IAP	CATACCTGGCTCTGTCCAAGA	CGCTCCACCAACTAAGAACG	[212]
β-actin	TCACCCACACTGTGCCCATCTACGA	CAGCGGAACCGCTCATTGCCAATG	[211]
